# Hereditary Nonsyndromic Gingival Fibromatosis: Report of Family Case Series

**DOI:** 10.1155/2013/835989

**Published:** 2013-09-26

**Authors:** Syed Wali Peeran, Karthikeyan Ramalingam, Syed Ali Peeran, Marei Hamed Mugrabi, Khaled Awidat Abdulla

**Affiliations:** ^1^Department of Periodontology and Oral Implantology, Faculty of Dentistry, Sebha University, Sebha, Libya; ^2^Department of Oral Pathology & Microbiology, Faculty of Dentistry, Sebha University, Sebha, Libya; ^3^Department of Oral and Maxillofacial Prosthodontics, Faculty of Dentistry, Jazan University, Jazan 45 142, Saudi Arabia; ^4^Department of Periodontics, Faculty of Dentistry, Arab Medical University, Benghazi, Libya; ^5^Department of Oral Biology and Orthodontics, Faculty of Dentistry, Sebha University, Sebha, Libya

## Abstract

Hereditary gingival fibromatosis (HGF) is a rare, benign disorder with slowly progressive enlargement of maxillary and mandibular gingiva. Herewith, we report the first case series of HGF presenting among mother and all of her 3 children. Their complaints included unaesthetic appearance due to gingival growth, malocclusion, and difficulty in mastication. Conventional gingivectomy with oral hygiene measures and regular followup is the treatment of choice for such presentation.

## 1. Introduction

Hereditary gingival fibromatosis (HGF) is a rare type of gingival enlargement. HGF can present in two forms, a nodular form affecting the dental papillae or a symmetric uniform gingival enlargement. HGF is seen as pale-pink enlargements, firm and leathery in consistency [1].

The literature search in PubMed revealed only two case series on HGF [2, 3]. Herewith, we report the first case series to the best of our knowledge, comprising 4 patients with HGF involving the mother and all of her three children including 2 daughters and 1 son.

## 2. Case Presentation

A 14-year-old female was reported with her father to the Outpatient Department, Faculty of Dentistry, Sebha University, Sebha, Libya. Her chief complaint was gingival swellings in the posterior region of upper and lower jaws since her childhood. Previous medical and surgical histories were noncontributory. Family history revealed that similar presentations were seen in her elder sister, elder brother, and her mother. Her father was unaffected.

**Figure 1:**
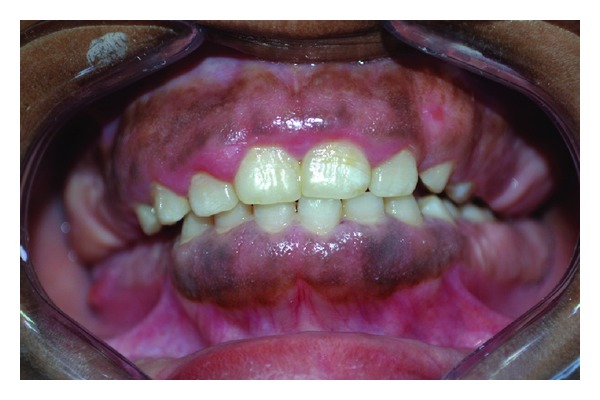
Clinical picture of 14-year-old female with gingival enlargement.

**Figure 2:**
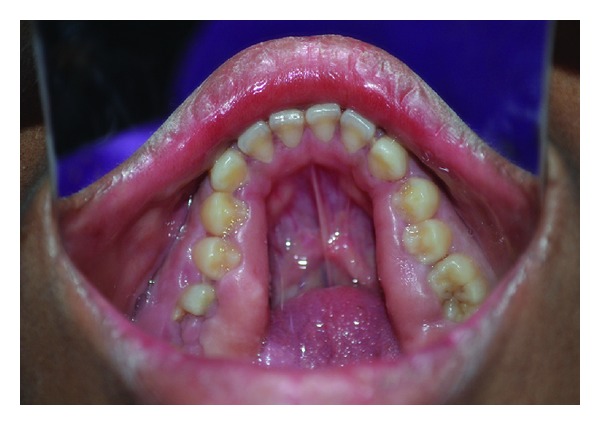
Clinical picture showing prominent enlargement in the posterior region.

**Figure 3:**
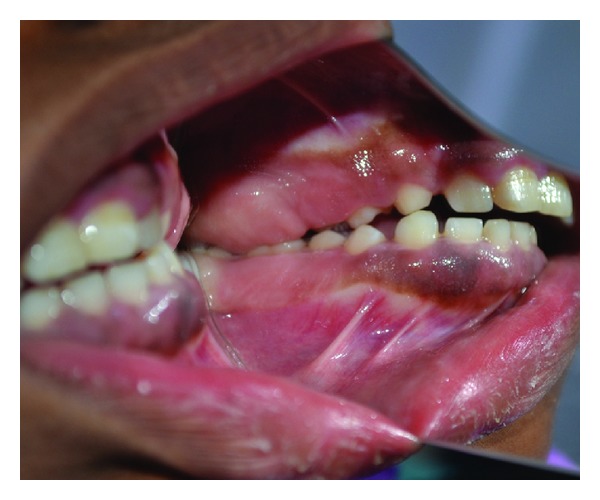
Clinical picture showing enlargement covering the crowns of molars.

**Figure 4:**
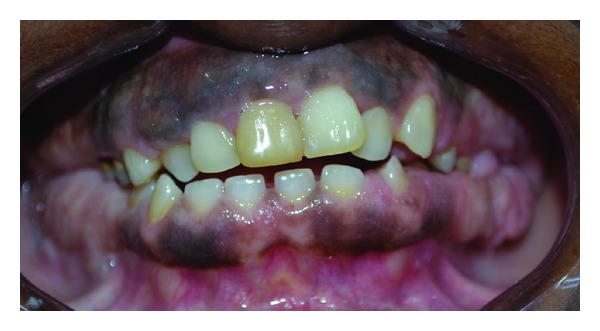
Clinical picture of 25-year-old sister with gingival enlargement.

**Figure 5:**
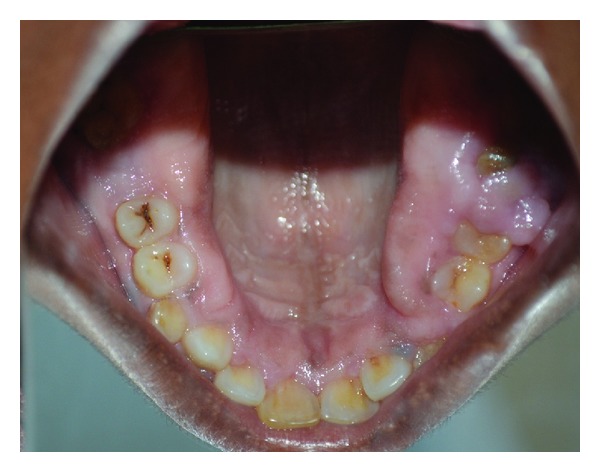
Clinical picture showing prominent gingival enlargement in posterior region.

**Figure 6:**
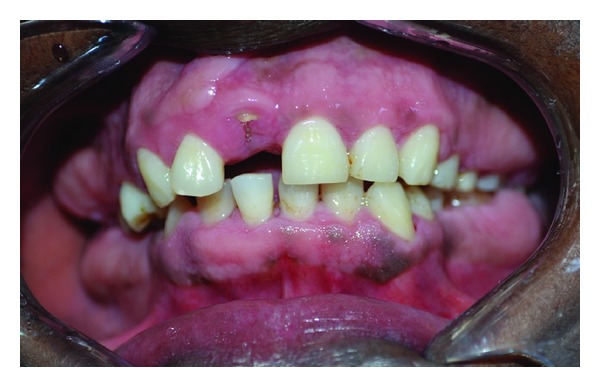
Clinical picture of 35-year-old brother with gingival enlargement.

**Figure 7:**
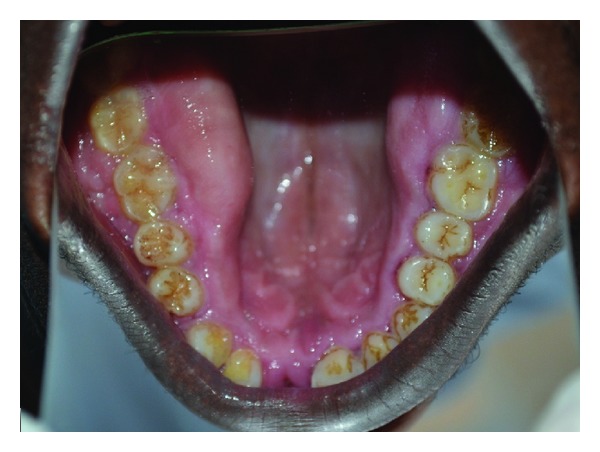
Clinical picture showing prominent enlargement in posterior region.

**Figure 8:**
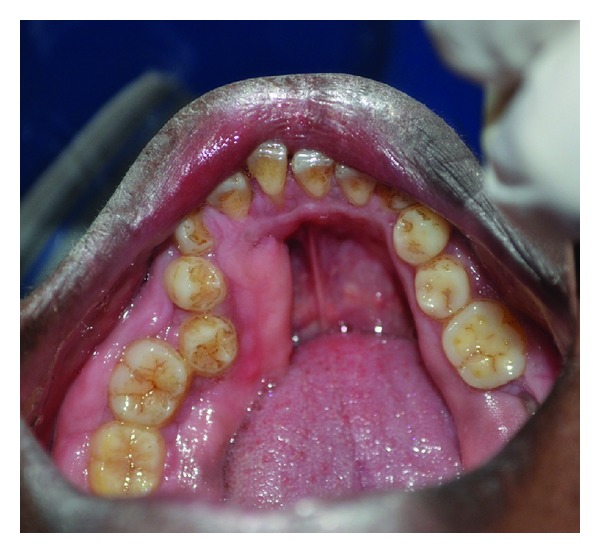
Clinical picture showing gingival enlargement and malocclusion.

**Figure 9:**
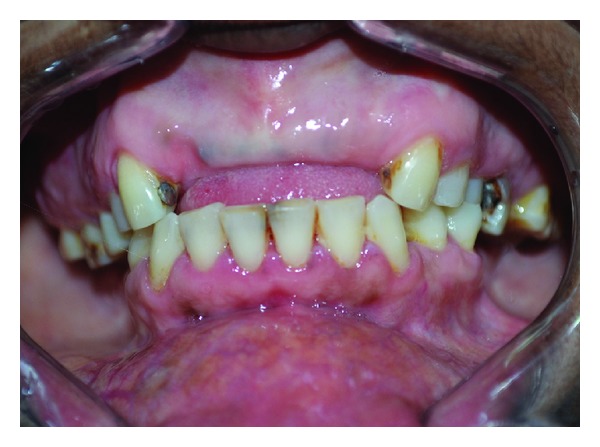
Clinical picture of 62-year-old mother with missing teeth.

**Figure 10:**
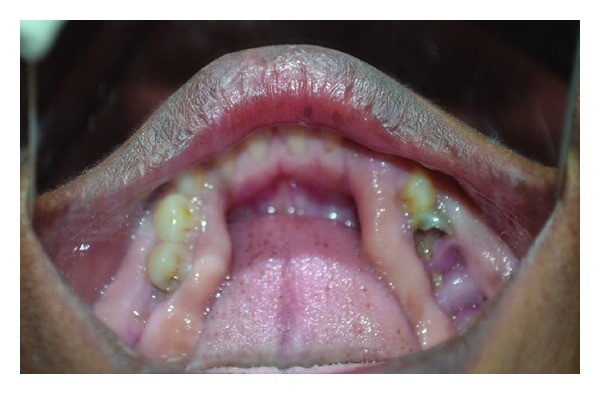
Clinical picture showing gingival enlargement in posterior region.

Clinical examination revealed diffuse, firm gingival enlargements symmetrically involving the maxillary and mandibular gingiva bilaterally (Figure 1). The enlargements were more severe in the molar region (Figure 2) and crowns were covered by soft tissue (Figure 3). Her family members were requested to report for clinical examination. Her elder sister was 25 years of age. She also had bilateral, symmetrical, and gingival enlargements in the posterior region of maxillary and mandibular gingiva (Figure 4). The teeth were displaced from their normal position. She also had retained deciduous teeth and multiple grossly decayed teeth (Figure 5). Her elder brother was 35 years of age. He also had bilateral, symmetrical enlargements, more prominent in the molar region (Figure 6). He also had severe malocclusion with arch collapse (Figures 7 and 8). Her mother was 62 years of age. She gave a history of similar enlargements in her gingiva and underwent multiple gingival surgeries for the same reason. Clinical examination revealed multiple missing teeth and remaining roots (Figure 9), and gingival enlargement was still evident in the molar region of mandible (Figure 10).

Correlating the family history and clinical presentations, it was diagnosed as hereditary nonsyndromic gingival fibromatosis.

The patients were advised for gingivectomy and regular oral hygiene maintenance with followup as treatment plan.

## 3. Discussion

HGF occurs due to congenital or hereditary causes. Studies reveal an autosomal dominant inheritance with chromosomal abnormality in 2p21-p22 and 5q13-q22 [1, 4]. It could arise due to nutritional and hormonal factors [5]. A mutation in the *Son of Sevenless-1* (*SOS-1*) gene has been suggested as a possible cause. Our case series also suggests autosomal dominant inheritance with HGF affecting the mother and all her children, whereas the father is unaffected. 

HGF can present as a nonsyndromic isolated variant, occasionally with epilepsy, mental retardation, or hypertrichosis. HGF can also be associated with syndromes like Cowden's syndrome (multiple hamartomas), Zimmerman-Laband syndrome (defects of bone, nail, ear, nose, and splenomegaly), Murray-Purelie Drescher syndrome (multiple dental hyaline tumors), Rutherford syndrome (corneal dystrophy), Jones syndrome (sensori-neural deafness), and Cross syndrome (hypopigmentation with athetosis) [1, 6]. As our patients did not have any other manifestations, it can be diagnosed as the non-syndromic, idiopathic variant of HGF. 

The gingival enlargement does not occur until the eruption of the primary or permanent dentition [5]. It affects interdental papillae and marginal and attached gingiva unlike drug induced enlargement in which attached gingiva is uninvolved. The severity may vary from mild involvement of one quadrant to severe involvement of all four quadrants [4]. The enlarged tissues can partially or totally cover the crowns, cause diastemas and pseudopockets, delay tooth eruption, and cause malocclusion. It can also present with abnormal swallowing pattern, difficulty in speech, and mastication. Our case series correlated with the literature in the clinical features of HGF.

The precise mechanism of HGF is unknown but could be confined to the fibroblasts in the gingivae. The continuing recurrence of the enlargement after repeated surgery and a permanent remolding of tissues after extraction of teeth suggest the importance of the presence of teeth and the gingival crevice environment in the pathogenesis of such enlargements [7]. Some authors report an increase in the proliferation of gingival fibroblasts, whereas others report slowerthan normal growth. Increased collagen synthesis rather than decreased levels of collagenase activity may be involved [8]. In our case series, gingival overgrowth has subsided in areas of prior extraction.

Histopathology of affected tissue reveals dense connective tissue rich in coarse connective tissue fibres, with young fibroblasts and scarce blood vessels. Small calcified particles, amyloid deposits, osseous metaplasia, and islands of odontogenic epithelium may be seen unusually. The overlying epithelium is of variable thickness and can show hyperkeratosis with elongated rete ridges [9].

The finest and suggested treatment modality for HGF is gingivectomy. The literature reports high recurrence rate after surgery and required a close followup. There is debate regarding the time of surgery. Eruption of complete set of permanent teeth is the recommended time for surgery [10]. The mother has previously undergone multiple gingivectomies and remained without recurrence. The treatment plan advised for the other affected patients is gingivectomy with regular oral hygiene measures and frequent followup.

To conclude, herewith we report the first case series to the best of our knowledge of HGF affecting the mother and all her children. A multidisciplinary management is advised to completely rehabilitate the patient.
